# Diagnostic Value Investigation and Bioinformatics Analysis of miR-31 in Patients with Lymph Node Metastasis of Colorectal Cancer

**DOI:** 10.1155/2019/9740475

**Published:** 2019-12-16

**Authors:** Wu-wen Zhang, Xin-liang Ming, Yuan Rong, Chao-qun Huang, Hong Weng, Hao Chen, Jun-mei Bian, Fu-bing Wang

**Affiliations:** ^1^Department of Laboratory Medicine, Zhongnan Hospital of Wuhan University, Wuhan 430071, China; ^2^Department of Gastrointestinal Surgery, Hubei Key Laboratory of Tumor Biological Behaviors & Hubei Cancer Clinical Study Center, Wuhan Clinical Research Center for Peritoneal Carcinomatosis, Zhongnan Hospital of Wuhan University, Wuhan 430071, China; ^3^Center for Evidence-Based and Translational Medicine, Zhongnan Hospital of Wuhan University, Wuhan 430071, China; ^4^Department of Pathology, Zhongnan Hospital of Wuhan University, Wuhan 430071, China; ^5^Department of Paediatrics, Tongren Hospital of Wuhan University, Wuhan 430074, China

## Abstract

Colorectal cancer (CRC) is one of the most frequent cancers occurring in developed countries. Distant CRC metastasis causes more than 90% of CRC-associated mortality. MicroRNAs (miRNAs) play a key role in regulating tumor metastasis and could be potential diagnostic biomarkers in CRC patients. This study is aimed at identifying miRNAs that can be used as diagnostic biomarkers for CRC metastasis. Towards this goal, we compared the expression of five miRNAs commonly associated with metastasis (i.e., miR-10b, miR-200c, miR-155, miR-21, and miR-31) between primary CRC (pCRC) tissues and corresponding metastatic lymph nodes (mCRC). Further, bioinformatics analysis of miR-31 was performed to predict target genes and related signaling pathways. Results showed that miR-31, miR-21, miR-10b, and miR-155 expression was increased to different extents, while miR-200c expression was lower in mCRC than that in pCRC. Moreover, we found that the level of both miR-31 and miR-21 was notably increased in pCRC when lymph node metastasis (LNM) was present, and the increase of miR-31 expression was more profound. Hence, upregulated miR-31 and miR-21 expression might be a miRNA signature in CRC metastasis. Moreover, we detected a higher miR-31 level in the plasma of CRC patients with LNM compared to patients without LNM or healthy individuals. With the bioinformatics analysis of miR-31, 121 putative target genes and transition of mitotic cell cycle and Wnt signaling pathway were identified to possibly play a role in CRC progression. We next identified seven hub genes via module analysis; of these, TNS1 was most likely to be the target of miR-31 and had significant prognostic value for CRC patients. In conclusion, miR-31 is significantly increased in the cancer tissues and plasma of CRC patients with LNM; thus, a high level of miR-31 in the plasma is a potential biomarker for the diagnosis of LNM of CRC.

## 1. Introduction

Colorectal cancer (CRC) is one of the most common malignancies and the fourth leading cause of cancer-related death worldwide [1]. However, although the development of early screening modalities and adjuvant chemotherapies has improved the prognosis of CRC, advanced CRC with lymph node metastasis (LNM) remains to have extremely poor prognosis. LNM is common among patients with advanced CRC, and given the poor prognosis, a more sensitive and specific method for LNM diagnosis can significantly benefit the therapeutic planning and clinical follow-up for CRC patients [2]. Although several gene products seem to contribute to the malignancy of CRC, accurate predictive factors of the prognosis and recurrence of CRC are yet to be identified.

Among the gene products and signaling molecules involved in the development of CRC, microRNAs (miRNAs), which are short noncoding RNAs measuring 18–25 nucleotides in length, might serve as molecular targets for both diagnosis and therapy. miRNAs have been demonstrated to contribute to a new mechanism of regulating gene expression and are involved in various biological processes of human cancers [3]. They can regulate gene expression posttranscriptionally, and bioinformatics analysis has suggested that miRNAs are capable of regulating the expression of numerous mammalian genes, among which tumor-promoting genes and tumor suppressor genes are included [4]. miRNAs were reported to be active in cancer development, acting as oncogenes (e.g., miR-155 and miR-21), tumor suppressors (e.g., miR-15a and miR-16-1), or metastasis promoters (e.g., miR-10b, miR-182, and miR-29a) [5]. Aberrant miRNA expression is associated with human cancers, and thus miRNA profiling, as one of the most modern modalities for molecular characterization of tumors, is used for cancer diagnosis and prognostic prediction [6, 7].

Studies have suggested miRNA profiles in distinguishing CRC tissues from normal colorectal mucosa. Luo et al. have observed that 164 miRNAs were aberrantly expressed in CRC [8]. Further, miR-31 and miR-20a were reported to be significantly elevated, whereas miR-145 and miR-143 were significantly downregulated in CRC tissues [9–11]. In addition, several studies have further described the correlations between dysregulated miRNA expression levels with tumor features. For example, the expressions of miR-21, miR-31, and miR-20a were positively correlated with the expression levels of histological markers (Ki-67 and CD34) in CRC tissues [12]. miR-145 was negatively correlated with the expression of K-ras gene, while miR-21 was positively correlated with the expression of K-ras gene in CRC [13]. Results of these studies indicated that dysregulated miRNAs were involved in cell proliferation and angiogenesis in CRC development. However, few studies have focused on the relationship between miRNAs and tumor stages. miRNAs might influence migration, invasion, and intravasation of tumor cells and act as metastasis promoters in breast cancer [14, 15], urothelial carcinomas, melanoma, and CRC [16]. Interestingly, upregulation of miR-21 and miR-10b correlates with LNM of breast cancer, while downregulation of miR-31, miR-335, and miR-126 is associated with tumor recurrence [16]. Eslamizadeh et al. have investigated the diagnostic value of miRNA profile in CRC. According to their study, the plasma levels of miR-21, miR-31, miR-20a, and miR-135b were rising with the higher stages of CRC. By contrast, miR-145, miR-let-7g, and miR-200c were decreasing with the higher stages of CRC. And the expression levels of plasma miR-21, miR-31, and miR-135b were significantly different between patients with stage II and III CRC [17].

miRNAs might promote or inhibit tumor metastasis by regulating the expression of target genes [18]. miR-31 was found to target NF-*κ*B-inducing kinase (NIK) to negatively regulate the noncanonical NF-*κ*B pathway, and loss of miR-31 therefore triggers oncogenic signaling in adult T cell leukemia [19]. Another study on head and neck carcinoma suggested that miR-31 could activate hypoxia-inducible factor to promote cancer progression by targeting factor-inhibiting hypoxia-inducible factor [20]. Besides, the tumor suppressor gene RhoBTB1 was also suggested to be regulated by miR-31, which was associated with the progression of human colon cancer [21]. However, conclusive studies on the target genes and pathways in CRC are relatively rare. To further explore the possible mechanism by which miR-31 regulates CRC, we performed bioinformatics analysis on miR-31 to predict target genes and signaling pathways. And a comprehensive approach was used to screen for the most possible target genes, including GO and KEGG enrichment analysis, PPI network construction, validation of expression levels, correlation analysis, and survival analysis of the target genes.

The aim of this study was to (1) identify miRNAs that can be biomarkers for CRC metastasis. Towards this goal, we compared the expression of five miRNAs commonly associated with metastasis (i.e., miR-10b, miR-200c, miR-155, miR-21, and miR-31) between primary CRC (pCRC) tissues and corresponding metastatic lymph nodes (mCRC). Further, we aimed to (2) determine the mechanism by which miR-31 regulates CRC metastasis and thus adopted bioinformatics analysis to explore putative target genes and related pathways involved in CRC. Finally, we sought to (3) identify the most possible key target genes of miR-31 in CRC using comprehensive bioinformatics methods.

## 2. Materials and Methods

### 2.1. Patients and Samples

All experimental procedures and pathological classification were approved by the Zhongnan Hospital Ethics Committee of Wuhan University and were performed following the International Union Against Cancer and American Joint Committee on Cancer TNM staging system for colon cancer established in 2003. Informed consent was obtained from each patient prior to sampling. Tumor tissues were collected from CRC patients after tumor resection at Zhongnan Hospital of Wuhan University. None of them received any preoperative treatment. We designed three sets of experiments. In set 1, 9 primary CRC (pCRC) tissue samples and corresponding mCRC of the same patients (stage III/IV CRC) were paired to identify metastasis-related miRNAs. Each lymph node was tested via hematoxylin and eosin staining to confirm the LNM. In set 2, pCRC tissue samples were collected from matched CRC patients with or without LNM to validate the candidate miRNAs identified in set 1. In set 3, before surgery or preoperative treatments, blood plasmas were collected from 28 CRC patients (stage III/IV) with LNM, 28 patients (stage I/II) without LNM, and 28 age- and sex-matched healthy volunteers. All tissues and plasmas were well preserved immediately after collection until RNA extraction.

### 2.2. RNA Extraction

Small RNAs from the plasma were extracted with the miRVana PARIS RNA isolation kit (Qiagen, Germany). Briefly, 250 *μ*l of plasma was thawed on ice followed by centrifugation at 14,000 rpm for 5 minutes to remove cell debris and organelles. We then lysed 150 *μ*l of supernatant with an equal volume of 2 × denaturing solution (Qiagen, Germany). To normalize the variations among the samples, 25 fmol of synthetic Caenorhabditis elegans miRNA cel-miR-39 (BioVendor, Europe) was added into each denatured sample during the RNA extraction [22]. Small RNAs were then purified following the manufacturer's protocol except that 45 *μ*l of nuclease-free water was used to elute small RNAs.

Total RNAs from the tumor tissues were extracted using TRIzol reagent (TaKaRa, Tokyo, Japan) following the manufacturer's protocol. The concentration and purity of total RNAs were measured using the Smart Spec Plus spectrophotometer (Thermo Scientific Inc., USA).

### 2.3. Real-Time PCR and Expression Analysis

Plasma RNA or 100 ng tissue RNA was polyadenylated and reverse transcribed into cDNA using the miScript Reverse Transcription kit (Qiagen, Germany). RT-PCR for each sample was performed in duplicates using the miScript SYBR Green PCR kit (Qiagen, Hilden, Germany) on a DNA Engine Opticon II system (Bio-Rad Laboratories, Inc., Hercules, CA, USA). The miRNA-specific primers were designed based on the miRNA sequences obtained from the miRBase database. Each amplification reaction was performed in a final volume of 20 *μ*l containing 1 *μ*l of cDNA, 0.25 mM of each primer, and 1 × SYBR Green PCR Master mix. At the end of PCR, the melting curve analysis and electrophoresis of the PCR products on 3.0% agarose gels were performed to determine the specificity of amplification. U6 snRNA was used as the internal control.

The quantification of mature miRNA was performed using BioRad CFX Manager (Bio-Rad Laboratories, Inc., Hercules, CA, USA). The cycle threshold (Ct) was defined as the cycle number when the fluorescent signal crossed the threshold in PCR.

(1) $$\begin{array}{c} \Delta \text{Ct}=\text{Ct}\left(\text{miRNA of interest}\right)-\text{Ct}\left(\text{internal control}\right)\\ \Delta \Delta \text{Ct}=\Delta \text{Ct}\left(\text{tumor specimen}\right)-\Delta \text{Ct}\left(\text{normal control}\right) \end{array}$$

The relative miRNA expression was normalized to U6 levels and calculated through the 2^-*ΔΔ*Ct^ method [23]. The data were presented as the fold changes of expression relative to normal tissues.

### 2.4. Identification of Metastasis-Related miRNAs in pCRC and mCRC

To identify miRNAs specific for metastatic CRC, miRNAs in nine matched pCRC tissues and corresponding mCRC were analyzed via RT-PCR. We first tested the expression of five miRNAs, i.e., miR-10b, miR-200c, miR-155, miR-21, and miR-31, because they have been suggested to closely associate with metastasis in multiple tumor types.

### 2.5. Prediction of Putative Target Genes of miR-31

Target genes of miR-31 were predicted using three online databases frequently used for miRNA target prediction: TargetScan Release 7.2, miRDB, and DIANA-microT web server v5.0 [24–26]. The overlapping parts of the possible target genes were considered as the putative target genes.

### 2.6. GO and KEGG Clustering Analysis of Putative Target Genes

Putative target genes were uploaded into the function annotation portal of The Database for Annotation, Visualization, and Integrated Discovery (DAVID), online bioinformatics resources for investigating the biological meaning of large gene lists [27]. Gene ontology (GO) analysis was utilized to investigate the possible roles of target genes involved in biological processes (BP), cellular components (CC), and molecular functions (MFs). Kyoto Encyclopedia of Genes and Genomes (KEGG) clustering analysis were adopted to map genes to a related pathway. A *p* value of <0.05 and a false discovery rate of <0.05 were applied to identify significant GO and KEGG items.

### 2.7. Construction of PPI Network and Module Analysis

To study the interaction network among functional proteins, we constructed the protein-protein interactive (PPI) network with the StringApp plugin in Cytoscape from the STRING database [28]. Cytoscape 3.51 is a public platform for establishing biomolecular interaction networks [29]. Further, the CytoHubba containing 12 predicted algorithms (Stress, Betweenness, DMNC, Degree, MNC, MCC, BottleNeck, EPC, Closeness, Radiality, EcCentricity, and ClusteringCoefficient) was used to identify hub genes that were most likely key target genes of miR-31.

### 2.8. Validation of the Expression of miR-31 and Hub Genes in Colorectal Cancer

To validate the expression of miR-31 and hub genes in CRC, we downloaded RNA expression data of GDC TCGA Colon Cancer and Rectal Cancer and controls from the UCSC Xena platform [30]. All the expression data have been normalized with log2 transformation. Then, the RNA counts of miR-31 and hub genes were extracted. Expression levels of miR-31 and hub genes were compared between colon cancer, rectal cancer, and controls with Student's *t*-test using GraphPad Prism 6.0 (GraphPad Software, Inc., La Jolla, CA, USA). The difference in miR-31 and hub gene expression was shown as a box plot. In addition, to reveal the relationship between miR-31 and the hub genes, Spearman's correlation analysis and linear regression plots construction were performed with GraphPad Prism 6.0.

### 2.9. Prognostic Value Evaluation of Hub Genes

To evaluate the value of these hub genes in CRC patients' prognosis, we used the GTEx survival data of colon and rectal cancer patients with the GEPIA online tool [31]. Overall survival (OS) and disease-free survival (DFS) of colon and rectal cancer patients were estimated by plotting Kaplan-Meier survival curves. Hazard ratio (HR) was calculated with the log-rank test to assess the effects of hub gene expression on patients' survival.

### 2.10. Statistical Analysis

All data were expressed as the mean ± SEM. Student's *t*-test and Mann-Whitney *U* test were applied to assess the statistical significance. To determine the extent to which the obtained -*Δ*Ct efficiently distinguishes different clinical subsets, the receiver operating characteristic (ROC) analysis was performed, and the area under curve (AUC) was used as an indicator of the distinguished capability. All statistical analyses were performed using SPSS 13.0 software, and *p* values below 0.05 were defined as statistically significant.

## 3. Results

### 3.1. Metastasis-Related miRNAs in pCRC and mCRC

The expressions of miR-10b, miR-155, miR-31, and miR-21 were higher, and the expression of miR-200c was lower in mCRC than that in corresponding pCRC tissues. These changes were observed in all nine cases tested. In particular, the expression of miR-21 and miR-31 in mCRC was both more than two-folds higher than those in pCRC tissues (Table 1). Therefore, we focused on miR-21 and miR-31 in further investigations to disclose their significance.

### 3.2. miR-21 and miR-31 Are Upregulated in CRC with LNM

**Figure 1:**
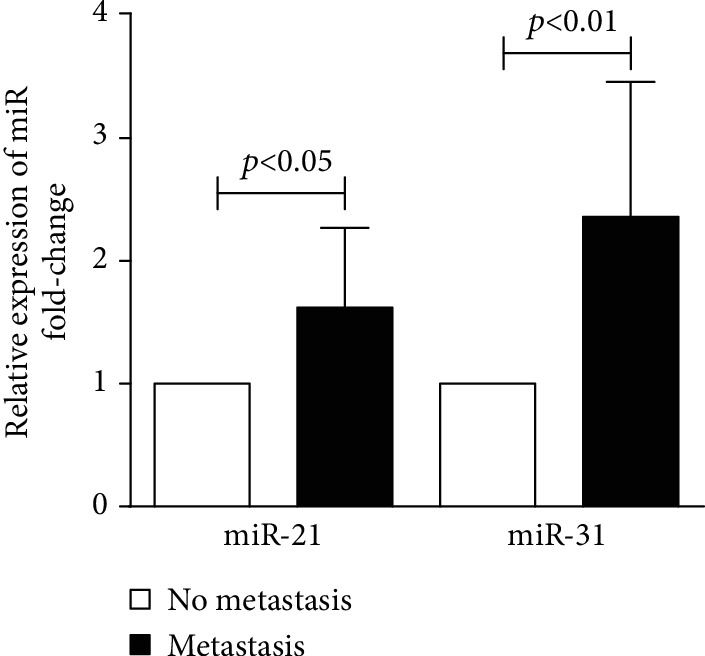
Relative expression of miR-21 and miR-31 in the pCRC samples collected from patients with or without LNM. *N* = 33 in each group.

To explore the value of miR-21 and miR-31 in the diagnosis of CRC metastasis, we assessed their expression in the pCRC tissues collected from patients with or without LNM via RT-PCR. Each group comprised 33 patients. The miR-21 level was significantly higher by 1.62-fold in average in pCRC tissues when LNM was present, as compared with pCRC tissues without LNM (*p* < 0.05) (Figure 1). Similarly, the miR-31 level was also considerably higher in the pCRC tissues of patients with LNM (average, 2.36-fold, *p* < 0.01).

### 3.3. Plasma miR-31 Distinguishes CRC Metastasis

**Figure 2:**
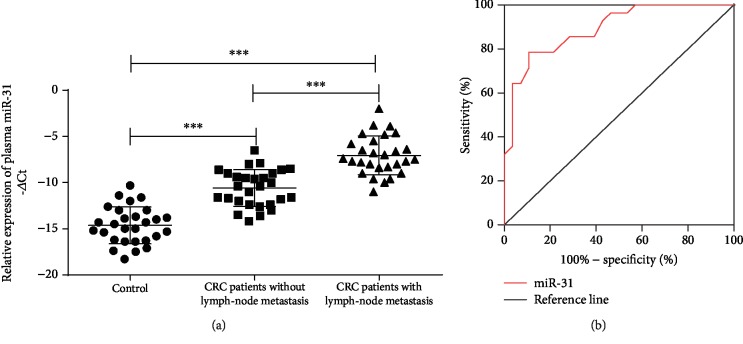
Plasma miR-31 distinguishes CRC metastasis. (a) Plasma miR-31 expression in healthy donors (*n* = 28), stage I/II CRC patients without LNM (*n* = 28), and stage III/IV CRC patients with LNM (*n* = 28). The -*Δ*Ct values were calculated via normalization to U6 snRNA expression. (b) Receiver operating characteristic (ROC) curve analysis using plasma miR-31 for distinguishing CRC metastasis. ∗∗∗ indicates *p* < 0.001.

To assess whether the upregulation of miR-31 can identify CRC or, more specifically, CRC metastasis, we determined the miR-31 level in the plasma of CRC patients and healthy donors. In total, 84 plasma samples were obtained from 28 stage I/II patients without LNM, 28 stage III/IV patients with LNM, and 28 healthy donors. The -*Δ*Ct value was used to indicate miR-31 expression in the plasma. Compared with that of healthy donors, CRC patients had significantly elevated miR-31 in the plasma regardless of LNM (*p* < 0.001). Furthermore, patients with LNM had even higher plasma miR-31 expression than patients without LNM (*p* < 0.001) (Figure 2(a)). The ROC curve revealed that miR-31 was potentially a valuable biomarker for discriminating CRC patients with LNM from CRC patients without LNM, as indicated by an AUC of 0.89 (95% CI: 0.81–0.97, *p* < 0.001) (Figure 2(b)). In the ROC assay, a -*Δ*Ct value of -8.6 (normalized) in patients with LNM was identified as a cut-off to discriminate metastatic CRC from nonmetastatic CRC. The optimal specificity and sensitivity were 86.2% and 78.5%, respectively (Figure 2(b)).

### 3.4. Putative Target Genes of miR-31

**Figure 3:**
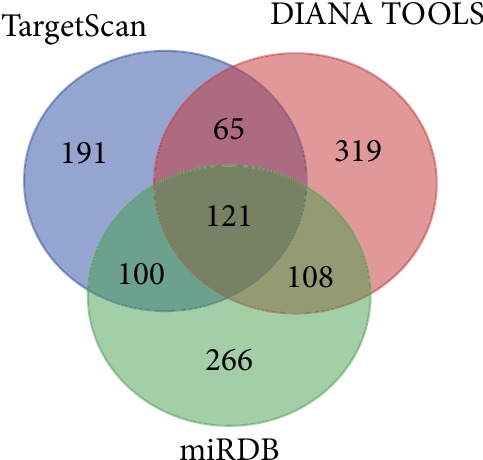
Target genes of miR-31 predicted by TargetScan, miRDB, and DIANA-microT web server. 121 overlapping genes of the three databases were considered putative target genes of miR-31.

There were 477, 613, and 595 target genes of miR-31 predicted using the TargetScan, miRDB, and DIANA-microT web server, respectively. The 121 overlapping genes of the 3 databases were considered target genes of miR-31 (Figure 3).

### 3.5. GO and KEGG Enrichment Analysis

**Figure 4:**
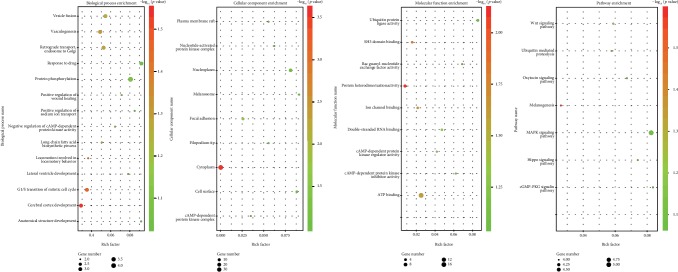
Putative target genes of miR-31 predicted by TargetScan, miRDB, and DIANA-microT web server. Overview of significantly enriched GO and KEGG terms. The *x*-axis represents the ratio of involved genes, and the *y*-axis represents the GO and KEGG terms. Each bubble represents a term. The size of the bubble indicates the number of involved genes. Lighter colors indicate smaller *p* values.

There were four, three, and six terms significantly clustered for BP, CC, and MF through GO analysis, respectively (Figure 4). The top three GO terms were cerebral cortex development, transition of mitotic cell cycle, and locomotion involved in locomotory behavior in BP; cytoplasm, focal adhesion, and cAMP-dependent protein kinase complex in CC; and protein heterodimerization activity, SH3 domain binding, and ion channel binding in MF. Seven pathways were identified via KEGG enrichment analysis (Figure 4), and the top three pathways were melanogenesis, ubiquitin-mediated proteolysis, and Wnt signaling pathway.

### 3.6. PPI Network and Hub Genes

**Figure 5:**
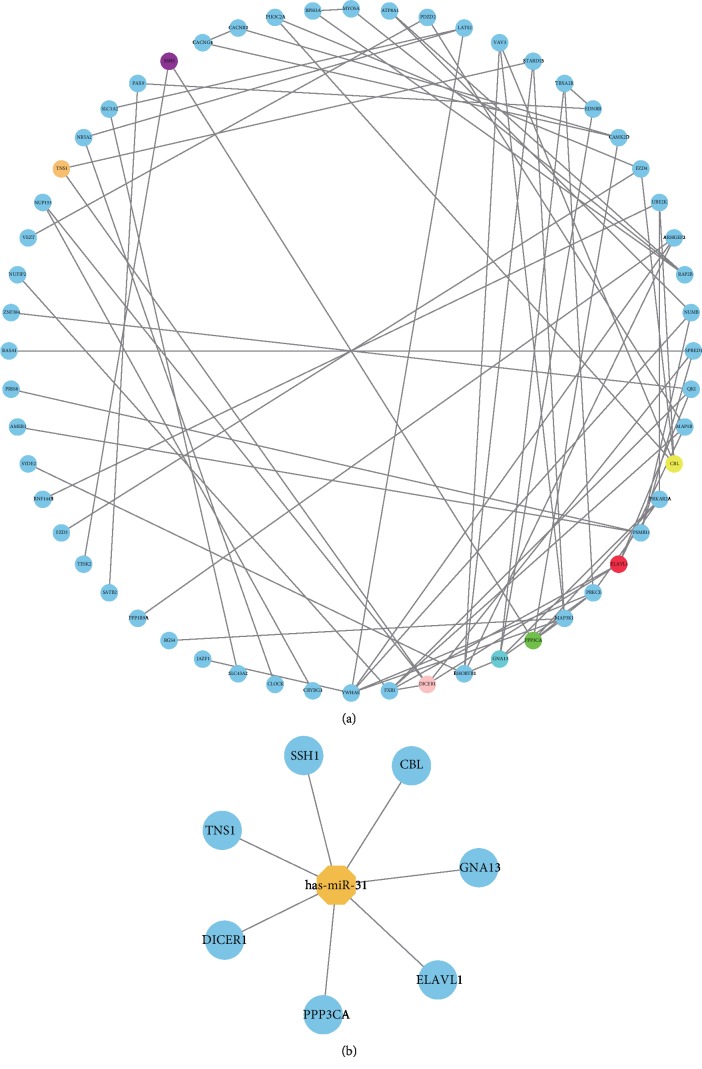
PPI network of possible miR-31 target genes and hub genes from the PPI network. (a) In the PPI network, each node represents a gene-encoded protein, while lines between the nodes represent protein associations. (b) Seven hub genes identified from the PPI network.

The PPI network was constructed with the 121 putative target genes using StringApp (Figure 5(a)). Each of the 121 target genes may associate with others, which may constitute interactive modules involved in the progression of CRC. After module analysis using 12 algorithms, the results were sorted in a descending order, and seven hub genes (ELAVL1, PPP3CA, DICER1, CBL, GNA13, SSH1, and TNS1) were identified, which are probably the key target genes of miR-31 (Figure 5(b)).

### 3.7. Validation of the Expression of miR-31 and Hub Genes

**Figure 6:**
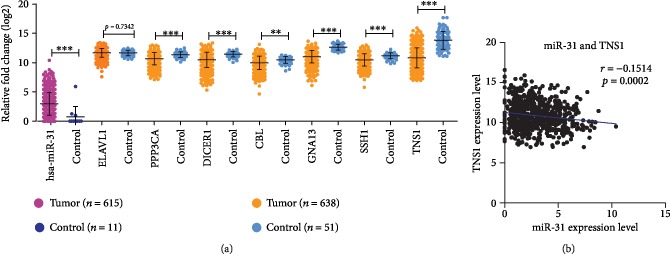
Validation of the expression of miR-31 and hub genes in CRC and controls tissues. (a) Expression of miR-31 and hub gene were detected in 615 CRC tissues and 11 control tissues. miR-31 was significantly upregulated in CRC tissues compared to control tissues. Expression of the hub genes was detected in 638 CRC tissues and 51 control tissues. Six hub genes—PPP3CA, DICER1, CBL, GNA13, SSH1, and TNS1—were significantly downregulated in CRC tissues compared to control tissues. No significant difference of ELAVL1 expression was observed between CRC tissues and control tissues. (b) Correlation analysis showed that TNS1 expression level was negatively correlated with miR-31 expression level in CRC and control tissues.

To compare the expression of miR-31 between CRC tissues and controls, miRNA expression data of 615 tumor tissues and 11controls was downloaded. The miR-31 level was significantly higher in CRC tissues than that in controls (Figure 6(a)). As for the hub genes, mRNA expression data of 638 GDC TCGA tumor tissues (including colon cancer and rectal cancer) and 51 controls were downloaded (Figure 6(a)). Except for ELAVL1, all of the other six hub genes were significantly downregulated in colon and rectal tumor tissues compared to those in controls. Considering that miR-31 was significantly upregulated in CRC, these six hub genes are possibly regulated by miR-31 in CRC. Further, correlation analysis showed that TNS1 level was negatively correlated with miR-31 (Figure 6(b)); meanwhile, no statistically significant relationships between the other six hub genes and miR-31 were found.

### 3.8. Prognostic Value Evaluation of Hub Genes

**Figure 7:**
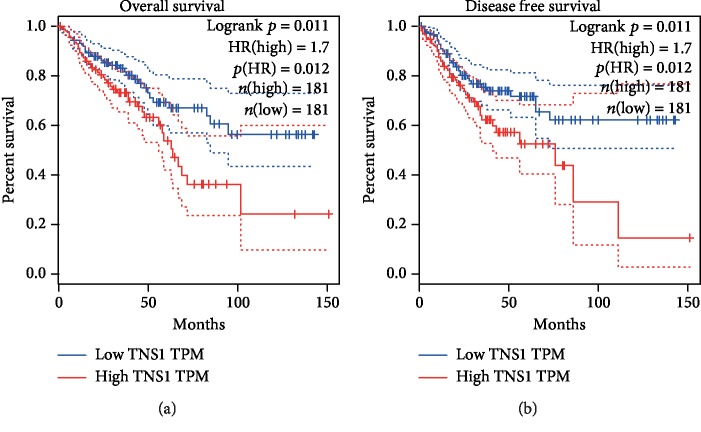
Kaplan-Meier survival curves of 362 CRC cases according to TNS1 expression levels. (a) OS survival curve of CRC patients based on TNS1 expression level (HR = 1.7, *p* = 0.012). (b) DFS survival curve of CRC patients based on TNS1 expression level (HR = 1.7, *p* = 0.012).

Data of 362 CRC patients were used in Kaplan-Meier survival analysis for seven hub genes. The results indicated that low TNS1 expression was significantly associated with improved OS (HR = 1.7, *p* = 0.012) and DFS (HR = 1.7, *p* = 0.012) (Figure 7). However, for the other six hub genes including ELAVL1, PPP3CA, DICER1, CBL, GNA13, and SSH1, no statistical significance was found in the survival analysis (Supplement Figures [Supplementary-material supplementary-material-1]–[Supplementary-material supplementary-material-1]). Further studies with larger cohorts need to be carried out.

## 4. Discussion

Amplification, deletion, and rearrangement of miRNAs are frequently present in human cancers. Some altered miRNA expression can promote tumorigenesis, and some miRNAs act as tumor suppressors [32]. Recently, the term “metastamiR” was proposed to describe miRNAs that are associated with tumor metastasis. MetastamiRs can be prometastatic or antimetastatic [16]. For example, miR-10b was firstly reported to contribute to breast cancer metastasis [33]. Later, it was reported that miR-335 could inhibit the invasion of metastatic breast cancer cell. Increasing evidence has demonstrated altered miRNA expression between primary and metastatic tumors, implying the important role of miRNAs in tumor metastasis. Previous studies have reported that 5 upregulated miRNAs and 14 downregulated miRNAs are involved in the metastasis of CRC, but the association between miRNA levels and CRC metastasis remains unclear [34, 35]. Thus, it is necessary to investigate the differential miRNA levels between primary tumors and metastatic tissues. A matched comparison method is optimal because it can exclude endogenous differences of miRNA expression. In our research, the levels of five metastasis-related miRNAs were detected and most of them were increased in mCRC. The trend of miRNA expression in our results was similar to previous investigations [33, 36, 37]. In our study, the higher expression of miR-31, miR-21, miR-10b, and miR-155 in mCRC indicated a miRNA signature might predict CRC metastasis. Because miR-31 and miR-21 were the most elevated miRNAs among the five selected miRNAs, we then wondered if they can be potential metastatic biomarkers of pCRC. As expected, their expression was significantly elevated in the pCRC with LNM. Interestingly, the increase of miR-31 was more profound, suggesting that miR-31 might be a more sensitive biomarker to predict CRC metastasis.

Although the alterations in miRNA expression in pCRC with LNM might be helpful for diagnosis, it is difficult for clinical practitioners to collect CRC tissues from patients. A more convenient and less invasive detection approach, such as blood testing, will be substantially beneficial for the prediction or diagnosis of CRC metastasis. Nucleic acid levels in the circulation can be used for the diagnosis of CRC [7, 38]. Previously, plasma miRNAs levels were reported to be highly correlated with miRNA expression in tumor tissue from breast cancer patients [39]. miRNAs have been detected in the serum and plasma of CRC, ovarian cancer, and prostate cancer patients. The plasma miRNAs are more stable and consistent than other circulating nucleic acids. Hence, they could be optimal biomarkers for cancer diagnosis. For example, increased plasma miR-92 levels can accurately discriminate CRC from gastric cancer and benign disease [40]. Plasma miR-141 has been proposed in diagnosing metastatic colon cancer [41]. We also evaluated the plasma miR-31 levels in CRC patients with or without LNM. Compared to CRC patients without LNM, patients with LNM have significantly higher plasma miR-31 level. miR-31 yielded a ROC curve area of 0.89 with a sensitivity of 78.5% and specificity of 86.2% in distinguishing CRC with LNM from CRC without LNM, using a cut-off value of -8.6 (normalized).

As far as we know, plasma miR-31 has been investigated as a biomarker for oral cancer [42]. Eslamizadeh et al. have reported the plasma miR-31 level was rising with the higher stages of CRC [17]. Here, we come to the conclusion that plasma miR-31 is of diagnostic value for CRC with LNM. However, to further validate our study, future investigations should recruit a large sample cohort.

To explore the associations of miR-31 with CRC metastasis, we predicted putative target genes of miR-31 and then carried out enrichment analysis of functions and signaling pathways of the target genes with bioinformatics methods. Results of enrichment analysis show that the target genes of miR-31 are possibly involved in some cancer-related biological process and signaling pathways such as G1/S transition of mitotic cell cycle, ATP binding, and Wnt signaling pathway. PPI network construction and module analysis were further conducted, and seven hub genes were identified, which were more likely to be target genes of miR-31. Through validation of expression and correlation analysis, among the seven hub genes, the TNS1 level was found lower in CRC tissues compared to controls and was negatively correlated with the miR-31 level. Thus, TNS1 is most possibly to be regulated by miR-31. Through survival analysis, we found that the TNS1 level was significantly associated with OS and DFS of CRC patients. In addition, low expression of TNS1 was predictive of improved OS and DFS for CRC patients.

TNS1 is a 220 kD protein localized to focal adhesions and regions of the plasma membrane where the cell attaches to the extracellular matrix. TNS1 protein plays a role in regulating cell motility and is suggested to be involved in tumorigenesis [43]. Elevated TNS1 levels were associated with a poor overall survival in CRC patients. Therefore, we suspect miR-31 may target TNS1, contributing to improved outcomes for CRC patients. Further experimental studies need to be performed to validate whether TNS1 is actually targeted by miR-31 in CRC.

## 5. Conclusion

In summary, miR-31 is significantly elevated in tumor tissues and plasma of CRC patients with LNM. Plasma miR-31 may be utilized as a biomarker for CRC with LNM. In addition, elevated miR-31 may contribute to improved outcomes for CRC patients by targeting TNS1.

## Figures and Tables

**Table 1:** The expression of indicated miRNAs in mCRC relative to those in pCRC^∗∗^.

miR name	Fold change	Cases/all cases	Dysregulation
miR-10b	1.85	9/9	Upregulation
miR-155	1.22	9/9	Upregulation
miR-21^∗^	2.45	9/9	Upregulation
miR-31^∗^	3.12	9/9	Upregulation
miR-200c	0.65	9/9	Downregulation

^∗∗^The expression of each miRNA in pCRC was set as 1.

## Data Availability

The data used to support the findings of this study are available from the corresponding author upon request.

## Supplementary Materials

Supplementary Materials Supplement Figure 1: Kaplan-Meier survival curves of 362 CRC cases according to ELAVL1 expression levels. Supplement Figure 2: Kaplan-Meier survival curves of 362 CRC cases according to PPP3CA expression levels. Supplement Figure 3: Kaplan-Meier survival curves of 361 CRC cases according to DICER1 expression levels. Supplement Figure 4: Kaplan-Meier survival curves of 360 CRC cases according to CBL expression levels. Supplement Figure 5: Kaplan-Meier survival curves of 362 CRC cases according to GNA13 expression levels. Supplement Figure 6: Kaplan-Meier survival curves of 362 CRC cases according to SSH1 expression levels.
