# Supplementary material for: Diagnostic Value Investigation and Bioinformatics Analysis of miR-31 in Patients with Lymph Node Metastasis of Colorectal Cancer
Source: Anal Cell Pathol (Amst). 2019 Dec 16;2019:9740475. doi: 10.1155/2019/9740475 (PMC6942701; doi:10.1155/2019/9740475)
Supplement: Supplementary Materials — Supplement Figure 1: Kaplan-Meier survival curves of 362 CRC cases according to ELAVL1 expression levels. Supplement Figure 2: Kaplan-Meier survival curves of 362 CRC cases according to PPP3CA expression levels. Supplement Figure 3: Kaplan-Meier survival curves of 361 CRC cases according to DICER1 expression levels. Supplement Figure 4: Kaplan-Meier survival curves of 360 CRC cases according to CBL expression levels. Supplement Figure 5: Kaplan-Meier survival curves of 362 CRC cases according to GNA13 expression levels. Supplement Figure 6: Kaplan-Meier survival curves of 362 CRC cases according to SSH1 expression levels. [file 9740475.f1.docx]

**Figure legends**

**Supplement Figure 1 Kaplan-Meier survival curves of 362 CRC cases according to ELAVL1 expression levels.** (A) OS survival curve of CRC patients based on ELAVL1 expression level (HR = 0.77, p = 0.24). (B) DFS survival curve of CRC patients based on ELAVL1 expression level (HR = 0.89, p = 0.6).

**Supplement Figure 2 Kaplan-Meier survival curves of 362 CRC cases according to PPP3CA expression levels.** (A) OS survival curve of CRC patients based on PPP3CA expression level (HR = 0.79, p = 0.28). (B) DFS survival curve of CRC patients based on PPP3CA expression level (HR = 0.87, p = 0.51).

**Supplement Figure 3 Kaplan-Meier survival curves of 361 CRC cases according to DICER1 expression levels.** (A) OS survival curve of CRC patients based on DICER1 expression level (HR = 1.1, p = 0.58). (B) DFS survival curve of CRC patients based on DICER1 expression level (HR = 0.91, p = 0.66).

**Supplement Figure 4 Kaplan-Meier survival curves of 360 CRC cases according to CBL expression levels.** (A) OS survival curve of CRC patients based on CBL expression level (HR = 0.87, p = 0.53). (B) DFS survival curve of CRC patients based on CBL expression level (HR = 1.0, p = 0.99).

**Supplement Figure 5 Kaplan-Meier survival curves of 362 CRC cases according to GNA13 expression levels.** (A) OS survival curve of CRC patients based on GNA13 expression level (HR = 0.82, p = 0.35). (B) DFS survival curve of CRC patients based on GNA13 expression level (HR = 0.98, p = 0.91).

**Supplement Figure 6 Kaplan-Meier survival curves of 362 CRC cases according to SSH1 expression levels.** (A) OS survival curve of CRC patients based on SSH1 expression level (HR = 0.88, p = 0.56). (B) DFS survival curve of CRC patients based on SSH1 expression level (HR = 1.0, p = 0.99).


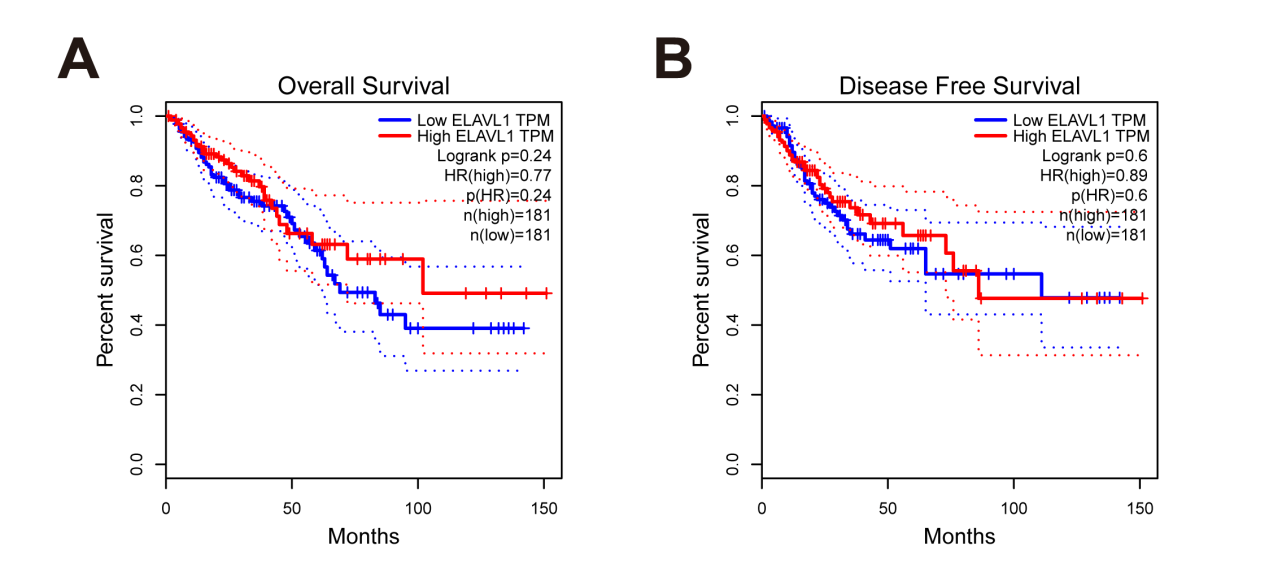


**Supplement Figure 1**

**
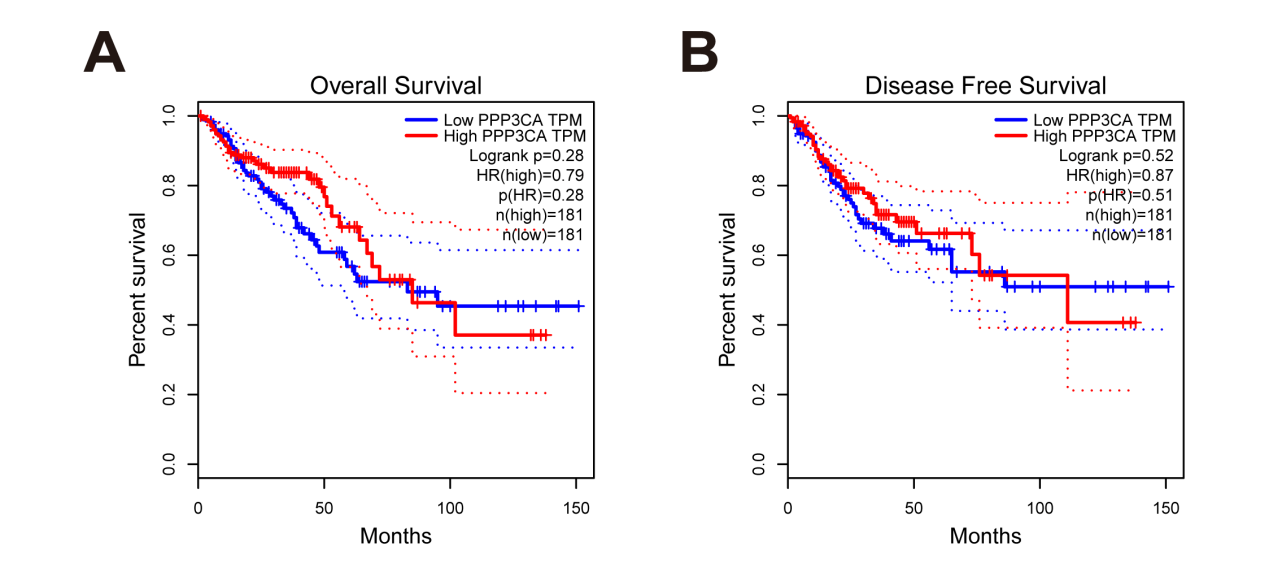
**

**Supplement Figure 2**

**
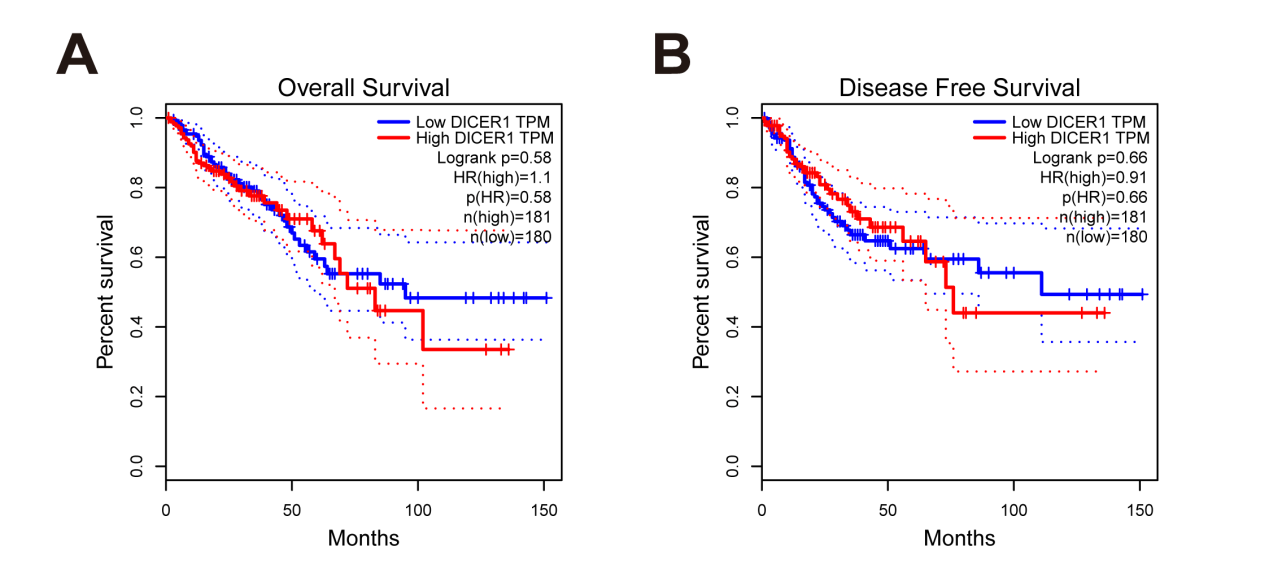
**

**Supplement Figure 3**


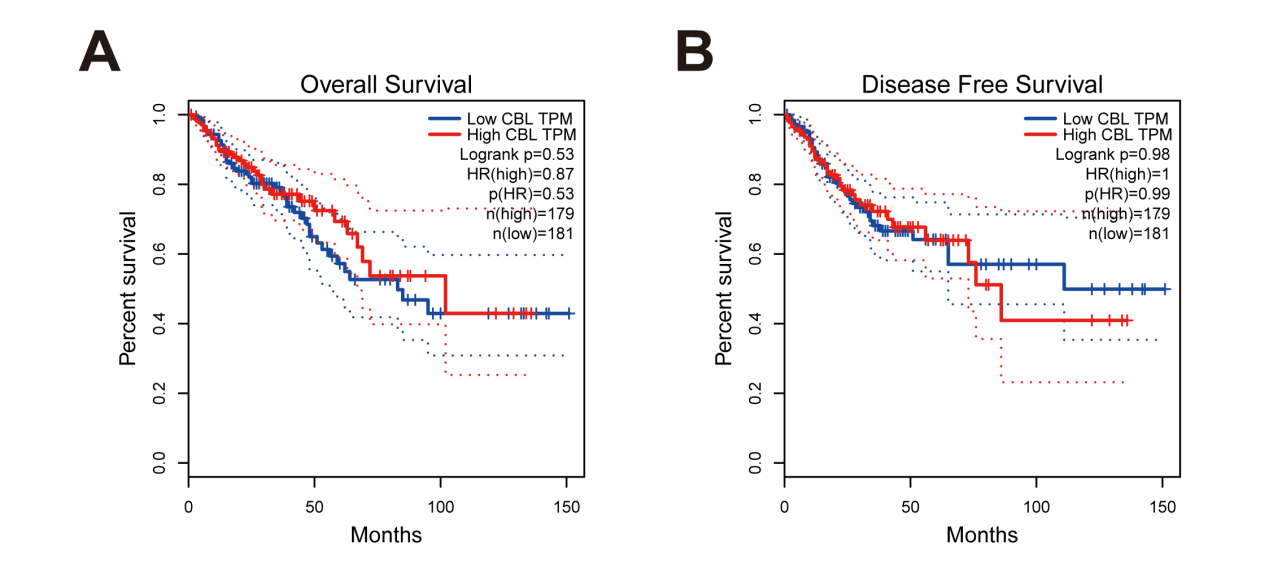


**Supplement Figure 4**


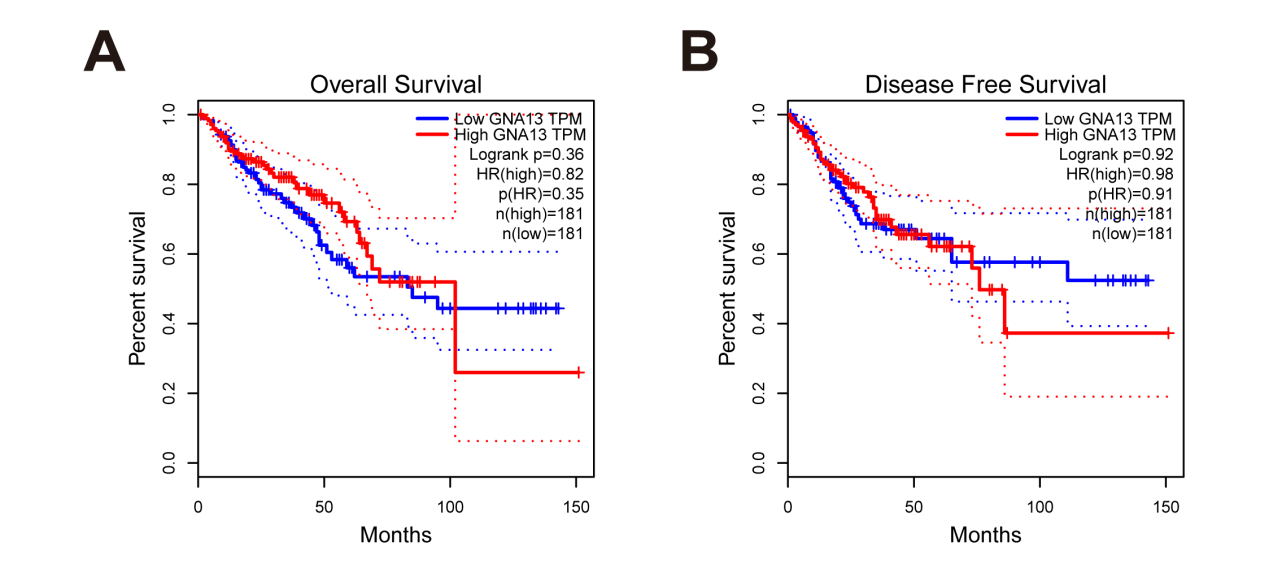


**Supplement Figure 5**


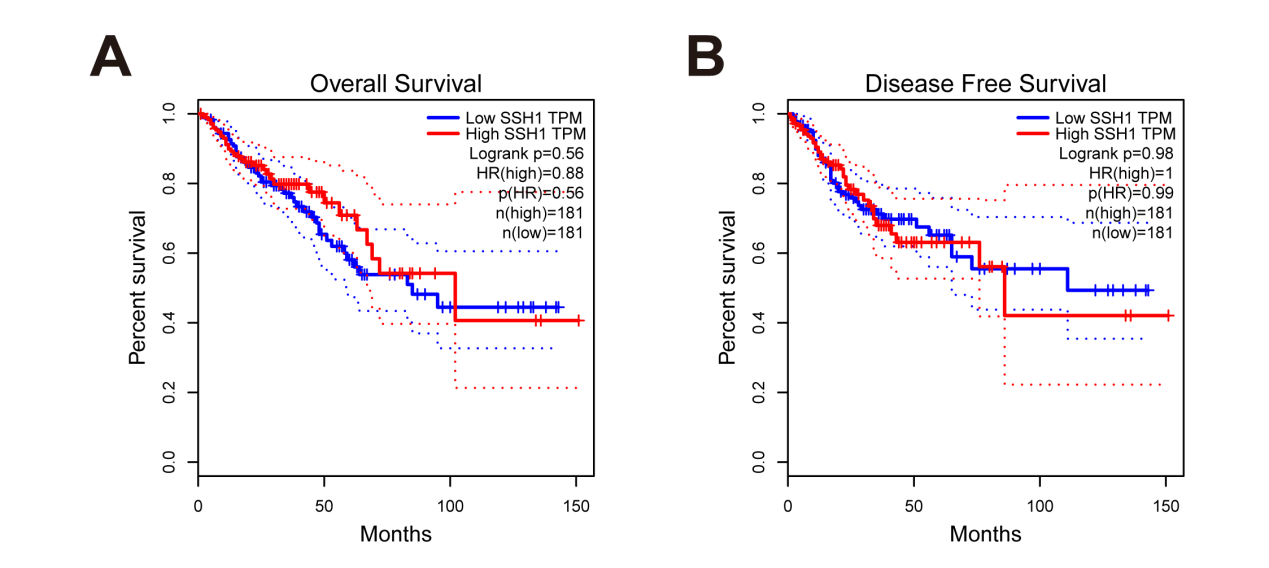


**Supplement Figure 6**
